# Life-Course Socioeconomic Position and Mild Cognitive Impairment in Midlife: Evidence from the 1958 British Birth Cohort

**DOI:** 10.1007/s44197-023-00173-6

**Published:** 2024-01-08

**Authors:** Chanthie Menika Sahota, Noriko Cable, Dorina Cadar

**Affiliations:** 1 Department of Epidemiology and Public Health, University College London, London, UK; 2 Department of Behavioural Science and Health, University College London, London, UK; 3 Department of Neuroscience, Brighton and Sussex Medical School, Trafford Centre, Sussex, BN1 9RH UK; 4 Department of Primary Care, Brighton and Sussex Medical School, Sussex, UK

**Keywords:** Mild cognitive impairment, Life-course socioeconomic position, NCDS, 1958 British birth cohort

## Abstract

**Background:**

Dementia has been the leading cause of death in the UK since 2015. Increasing evidence supports an association between socioeconomic position (SEP) and dementia onset in later life. However, limited studies have examined how life-course SEP influences the development of mild cognitive impairment (MCI), an intermediate state potentially preceding dementia. Therefore, the present study aims to examine the relationship between life-course SEP and MCI amongst adults aged 50 years in Great Britain.

**Methods:**

We employed data from the National Child Development Study (NCDS), also known as the 1958 British Birth Cohort, to determine the associations between SEP and MCI in 6590 participants. We categorised life-course measures of SEP as stable high/low or moving upward/downward over the life course. We assessed MCI at age 50 using one standard deviation below the averaged combined scores from all cognitive tests available. We then used binary logistic regression to estimate the longitudinal associations between life-course SEP and MCI.

**Results:**

Relative to those of a high SEP across the life course, participants who moved upward, downward, or remained at a low SEP were significantly associated with 25% (95% CI 1.02–1.54, *p* = 0.035), 70% (95% CI 1.27–2.27, *p* < 0.001), and 85% (95% CI 1.50–2.29, *p* < 0.001), respectively, higher odds of MCI, independent of all selected covariates.

**Conclusions:**

Lower life-course SEP was associated with significantly higher odds of MCI onset in middle life within the NCDS cohort. Public health policies targeting cognitive impairment should encompass a life-course approach to reduce socioeconomic inequalities.

**Supplementary Information:**

The online version contains supplementary material available at 10.1007/s44197-023-00173-6.

## Introduction

Affecting approximately 900,000 individuals living in the UK, dementia serves as a significant public health burden [1]. Current dementia care reportedly costs £34.7 billion and is expected to increase to £94.1 billion in 2040 as the aging population grows [1].

Several studies have identified an association between dementia and socioeconomic position (SEP), as composite factors of SEP may influence inequalities in dementia development [2–7]. Examining the effects of childhood and adult SEP, dementia symptoms and onset were 38% and 68%, respectively, significantly higher for elderly individuals in the lowest SEP category relative to those in the highest [2, 4].

However, there is a strong consensus that the pathology of dementia likely occurs much earlier than the emergence of its associated clinical features [8–11]. This is demonstrated by findings of an inverse association between cognitive impairment without dementia and SEP amongst older individuals in the current literature [12–15]. Therefore, mild cognitive impairment (MCI), which is an intermediate state in which cognitive decline is greater than that expected of normal aging but less than that of dementia and overall daily functioning is preserved, should be targeted for early intervention and dementia prevention [7, 16–20].

Furthermore, earlier forms of cognitive impairment, i.e., MCI, have rarely been explored in relation to SEP, especially in the UK, where dementia serves as a prominent and rising health burden. To address these research gaps, the present study used longitudinal data from the British National Child Development Study (NCDS) to examine how different life-course SEP backgrounds influence the onset of MCI amongst 50-year-old adults in this population.

## Methods

### Study Sample

**Fig. 1:**
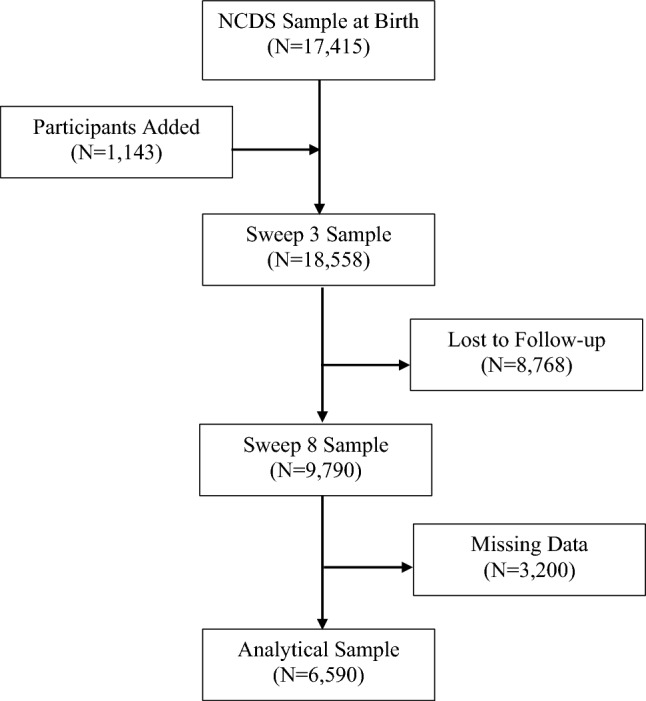
Flowchart representing the sample selection process using the 1958 British Birth Cohort

The National Child Development Study (NCDS), also known as the 1958 British Birth Cohort, was established using 98.7% (*N* = 17,415) of individuals born in the same week of March 1958 in England, Scotland, and Wales [21, 22], aiming to be representative of the British population. Since birth, this cohort has completed ten follow-ups, termed sweeps, to collect extensive information on socioeconomic resources and health. Additional details concerning data collection procedures can be found elsewhere [21, 22]. Immigrants born in the same reference week were added at Sweeps 1–3, increasing the study size to 18,558 participants (Fig. 1) [22]. Participants who were lost to follow-up or had missing data for at least one variable of interest were excluded from the analytical sample, resulting in an analytical sample size of 6590 participants. For the present analyses, we collected childhood data from birth to Sweep 2, while Sweeps 5, 6, and 8 were used for data in adulthood. The NCDS cohort received approval from the UK Multi-Centre Research Ethics Committee [21]. Written informed consent was obtained from parents and cohort members during childhood and adulthood, respectively.

### MCI

Cognitive performance data were collected at Sweep 8 when participants were 50 years. During this sweep, participants underwent cognitive test batteries administered via face-to-face computer-assisted personal interviews. The tests performed were Immediate Word Recall, Delayed Word Recall, Animal Naming, and Letter Cancellation, all of which are standard tests that have been frequently used to detect dementia and, more recently, MCI [9, 23]. The Immediate Word Recall test involved remembering a list of ten words immediately after hearing them, while Delayed Word Recall involved recalling the same list of words after a delay, which was at the end of the cognitive testing session [9]. The Animal Naming test required participants to name as many animals as they could within a specific period of time [9]. Finally, the Letter Cancellation test involved scanning a page with letters and identifying the number of P’s and W’s present in a timed manner. The present study used the accuracy score of the Letter Cancellation test only.

The Immediate Word Recall, Delayed Word Recall, and Animal Naming tests aid in assessing the verbal and semantic memory processing abilities of individuals [9]. The Letter Cancellation test represents visuospatial and attention skills [9]. Past studies have reported that the use of multiple cognitive domains to create a global score to detect MCI has greater diagnostic stability relative to assessments pertaining to solely one cognitive domain [24]. Thus, we combined the scores of each test to create a global cognitive function score ranging from 0 to 150. Scores less than one standard deviation (SD) below the global mean were classified as having MCI, while scores within one SD of the mean or greater were classified as not having MCI. A threshold of one SD is supported by the Diagnostic Statistical Manual-5 criteria in capturing individuals who may have MCI [25].

### Life-Course SEP

The present study used data pertaining to socioeconomic factors from various time points during birth, childhood, and adulthood to construct life-course SEP measures. Information about cohort members’ father’s social class at birth was predominantly used to assess SEP during childhood. If the information at birth was unavailable, data from Sweeps 1 and 2 when participants were 7 and 11 years, respectively, were used. In cases, where information about the father’s social class was unavailable, social class data of the participants’ maternal grandfather was used instead. We categorized childhood SEP as “high” (I professional–III non-manual) and “low” (III manual–V unskilled). SEP at adulthood was measured at Sweep 6 when participants were aged 42. Participants were asked about their current job at the time, which then determined their respective social class. We recategorized the data as “high” (I professional–III non-manual) and “low” (III manual–V unskilled). Participants, whose response was other were excluded from the analytical sample.

Both childhood and adulthood SEP data were used to create the life-course SEP variable, representing the SEP of participants from as close to birth to age 42 and potential changes in SEP that may have occurred during this time. This variable consisted of four categories: “always high”, “upward”, “downward”, and “always low”. The first category consisted of participants who were of a high SEP during both childhood and adulthood. The second category involved those who were of a low SEP during childhood but moved to a high SEP later in adulthood. In contrast, individuals who moved downward in the social hierarchy from a high to low SEP were assigned to the third category. Finally, the fourth category consisted of individuals with a low SEP at both time points.

### Covariates

The covariates selected by the present study were supported by previous research related to cognitive impairment and SEP in addition to the data collected from the NCDS cohort [7]. We selected data for all covariates at Sweep 6, except for education level and alcohol consumption, as these were ascertained at Sweeps 8 and 5, respectively. The demographic variables were sex, marital status, and education level. Education level was categorized based on the highest education level achieved at Sweep 8, by which “higher qualifications” included individuals with a diploma, degree or other degree level equivalent, or higher degree and “intermediate qualifications” consisted of anything less. The health behaviours measured were smoking status, physical activity, alcohol consumption, and fruit and vegetable intake. Data for alcohol consumption was collected at Sweep 5 when participants were aged 33 years. The health status covariates were the presence of a physical (ever had high blood pressure or diabetes) and mental health condition (measured using the 24-item Malaise Inventory) [26]. We scored each item of the Malaise Inventory and summed them to create a continuous variable, where higher scores indicated greater psychiatric distress.

### Statistical Analyses

We used four models of binary logistic regression to test the association between life-course SEP and MCI to determine the odds ratio (OR) and 95% confidence interval (CI) of MCI onset. The first model consisted of adjustments by sex and marital status. The second model was based on model 1, further adjusted for education level. In a similar manner, the third model consisted of the demographic variables and the health behaviour covariates. The fourth model consisted of the same covariates as the third model with the addition of the physical and mental health variables. We also conducted additional supplementary analyses with SEP at birth and age 42 to further investigate the potential effects SEP may have on MCI at these time points. We carried out all analyses using Stata MP 16.0.

### Sensitivity Analysis

A sensitivity analysis was conducted to evaluate selection bias that may affect the findings of the present study. The analysis compared the distribution of all the variables between the analytical and excluded samples to determine if there were statistically significant differences between the two samples.

## Results

As shown in Table 1, 37.2% of participants had a low SEP at birth, but 66.7% had a higher SEP by age 42, of which 36.7% had moved upward in the socioeconomic hierarchy. However, 30% of all participants also remained at a high SEP at both time points, while 26.2% of participants remained at a low SEP. The prevalence of MCI identified in the study sample was 14.1%. Overall, the study sample was comprised of an approximately equal number of males and females. The majority of individuals were married and achieved intermediate academic qualifications. In addition, about 97% of the study sample was ethnically White British or Irish (not shown). Most had also engaged in regular physical activity, drank alcohol on a weekly basis, consumed fruit and vegetables daily, and never smoked. Furthermore, a higher percentage of participants did not have a physical health condition, and the study sample altogether had a relatively low mean score on the Malaise Inventory scale, suggesting that very minimal psychiatric symptoms were present amongst cohort members overall.

**Table 1:** Descriptive characteristics of the analytical sample from the 1958 British Birth Cohort

Variable	Category	No MCI (*N* = 5661)	MCI (*N* = 929)	Total participants (*N* = 6590)
SEP at birth	High	2196 (38.8%)	252 (27.1%)	2448 (37.2%)
	Low	3465 (61.2%)	677 (72.9%)	4142 (62.9%)
SEP at age 42	High	3938 (69.6%)	456 (49.1%)	4394 (66.7%)
	Low	1723 (30.4%)	473 (50.9%)	2196 (33.3%)
Life-course SEP	Always high	1814 (32.0%)	165 (17.8%)	1979 (30.0%)
	Upward	2124 (37.5%)	291 (31.3%)	2415 (36.7%)
	Downward	382 (6.8%)	87 (9.4%)	469 (7.1%)
	Always low	1341 (23.7%)	386 (41.6%)	1727 (26.2%)
Sex	Male	2813 (49.7%)	546 (58.8%)	3359 (51.0%)
	Female	2848 (50.3%)	383 (41.2%)	3231 (49.0%)
Marital status	Married	4260 (75.3%)	673 (72.4%)	4933 (74.9%)
	Cohabiting	494 (8.7%)	83 (8.9%)	577 (8.8%)
	Single	404 (7.1%)	82 (8.8%)	486 (7.4%)
	Divorced/separated/widowed	503 (8.9%)	91 (9.8%)	594 (9.0%)
Education level	No qualifications	589 (10.4%)	217 (23.4%)	806 (12.2%)
	Intermediate qualifications	3482 (61.5%)	623 (67.1%)	4105 (62.3%)
	Higher qualifications	1590 (28.1%)	89 (9.6%)	1679 (25.5%)
Smoking status	Never	2743 (48.5%)	416 (44.8%)	3159 (47.9%)
	Ex-smoker	1507 (26.6%)	227 (24.4%)	1734 (26.3%)
	Current	1411 (24.9%)	286 (30.8%)	1697 (25.8%)
Physically active	No	1312 (23.2%)	281 (30.3%)	1593 (24.2%)
	Yes	4349 (76.8%)	648 (69.8%)	4997 (75.8%)
Alcohol consumption	Never/infrequently	1043 (18.4%)	199 (21.4%)	1242 (18.9%)
	Monthly	1136 (20.1%)	195 (21.0%)	1331 (20.2%)
	Weekly	3482 (61.5%)	535 (57.6%)	4017 (61.0%)
Fruit & veg intake	Daily	3123 (55.2%)	425 (45.8%)	3548 (53.8%)
	3–6 days/week	818 (14.5%)	74 (8.0%)	892 (13.5%)
	2 or less days/week	1627 (28.7%)	399 (43.0%)	2026 (30.7%)
	Never	93 (1.6%)	31 (3.3%)	124 (1.9%)
Physical health condition	No	5023 (88.7%)	810 (87.2%)	5833 (88.5%)
	Yes	638 (11.3%)	119 (12.8%)	757 (11.5%)
Malaise Inventory score	Mean (SD)	3.10 (3.05)	3.64 (3.62)	3.18 (3.14)

Specifically looking at the study sample in relation to their status for MCI, 72.9% of those identified as having MCI were of a low SEP at birth. However, this sample had almost equal proportions of individuals with a high or low SEP at age 42. While 31.3% of cohort members with MCI moved upward in SEP, 41.6% remained at a low SEP since birth. Furthermore, only 17.8% of respondents remained at a high SEP over the life course, which is lower than those without MCI (32%), and 9.4% moved down within the SEP hierarchy. In addition, most participants with MCI were male and had a slightly higher mean Malaise Inventory score than those identified as not having MCI. Participants with MCI were similar to those without MCI in terms of other characteristics. However, the proportion of cohort members with MCI had a slightly higher percentage of participants with no academic qualifications, were current smokers, and consumed fruit and vegetables two or fewer days/week compared to those without MCI.

The findings of the logistic regression models demonstrated strong evidence of an inverse association between life-course SEP and MCI (Table 2). Compared to individuals who remained at a high SEP from birth to age 42, those who moved upward in SEP were significantly associated with 1.25 (95% CI 1.01–1.53) times higher odds of developing MCI after adjusting for all other covariates (*p* = 0.035). Those who moved downward were significantly associated with 1.70 (95% CI 1.27–2.27) times greater odds of developing MCI compared to those who remained at a high SEP, controlling for all covariates (*p* < 0.001). Participants who remained at a low SEP were also significantly associated with higher odds of MCI onset (OR 1.85, 95% CI 1.49–2.28) than those who remained at a high SEP, adjusting for all covariates (*p* < 0.001). Across the four models, sex, education level, fruit and vegetable consumption, and psychiatric symptoms represented by the Malaise Inventory greatly confounded the primary association (*p* < 0.05 for each).

**Table 2:** Results of the logistic regressions for life-course SEP and MCI in midlife in the analytical sample from the 1958 British Birth Cohort

Variable	Model 1			Model 2			Model 3			Model 4		
	OR	95% CI	*p* value	OR	95% CI	*p* value	OR	95% CI	*p* value	OR	95% CI	*p* value
*Life-course SEP*												
Always high	1	–	–	1	–	–	1	–	–	1	–	–
Upward	1.55	1.27–1.89	< 0.001	1.29	1.05–1.59	0.014	1.26	1.02–1.54	0.031	1.25	1.01–1.53	0.037
Downward	2.44	1.84–3.23	< 0.001	1.77	1.33–2.37	< 0.001	1.71	1.28–2.29	< 0.001	1.70	1.27–2.27	< 0.001
Always low	3.08	2.53–3.75	< 0.001	1.99	1.61–2.45	< 0.001	1.85	1.50–2.29	< 0.001	1.85	1.49–2.28	< 0.001
*Sex*												
Male	1	–	–	1	–	–	1	–	–	1	–	–
Female	0.76	0.66–0.88	< 0.001	0.75	0.65–0.87	< 0.001	0.74	0.63–0.87	< 0.001	0.72	0.61–0.84	< 0.001
*Marital status*												
Married	1	–	–	1	–	–	1	–	–	1	–	–
Cohabiting	1.04	0.81–1.33	0.760	1.03	0.80–1.32	0.831	1.02	0.79–1.31	0.892	1.02	0.79–1.31	0.897
Single	1.29	1.00–1.66	0.053	1.32	1.02–1.71	0.038	1.29	0.99–1.68	0.056	1.28	0.99–1.66	0.064
Divorced/sep/wid	1.12	0.88–1.43	0.355	1.07	0.84–1.37	0.566	1.05	0.82–1.34	0.720	1.04	0.81–1.33	0.752
*Education level*												
No quals				1	–	–	1	–	–	1	–	–
Intermediate quals				0.58	0.48–0.70	< 0.001	0.61	0.51–0.74	< 0.001	0.63	0.52–0.75	< 0.001
Higher quals				0.22	0.16–0.29	< 0.001	0.25	0.18–0.33	< 0.001	0.25	0.19–0.34	< 0.001
*Smoking status*												
Never							1	–	–	1	–	–
Ex-smoker							0.90	0.75–1.08	0.264	0.89	0.75–1.07	0.213
Current							0.92	0.77–1.10	0.364	0.91	0.76–1.09	0.307
*Physically active*												
No							1	–	–	1	–	–
Yes							0.85	0.72–1.00	0.044	0.86	0.73–1.01	0.058
*Alcohol consumption*												
Never/infrequently							1	–	–	1	–	–
Monthly							0.97	0.78–1.21	0.777	0.98	0.79–1.23	0.882
Weekly							0.83	0.69–1.01	0.058	0.85	0.70–1.02	0.087
*Fruit & veg intake*												
Daily							1	–	–	1	–	–
3–6 days/week							0.67	0.51–0.87	0.002	0.66	0.51–0.86	0.002
2 or less days/week							1.41	1.20–1.66	< 0.001	1.38	1.18–1.62	< 0.001
Never							1.67	1.08–2.58	0.022	1.61	1.04–2.50	0.033
*Physical health cond*												
No										1	–	–
Yes										1.05	0.85–1.31	0.639
Malaise Inventory score										1.03	1.01–1.06	0.002

Model 1 adjusted for sex and marital status; Model 2 adjusted for Model 1 covariates and education level; Model 3 adjusted for Model 2 covariates, smoking status, physical activity, alcohol consumption, and fruit and vegetable intake; Model 4 adjusted for Model 3 covariates, physical health conditions, and psychiatric symptoms

Independently assessing the effect of SEP at different time points on MCI formation, participants with a low SEP at birth (Supplementary Table 1) and age 42 (Supplementary Table 2) were significantly associated with 1.27 (95% CI 1.08–1.50) and 1.57 (95% CI 1.35–1.84), respectively, greater odds of MCI onset in later life relative to those born into a high SEP across all four models of adjustment (*p* = 0.003 and *p* < 0.001, respectively). As reported for the relationship involving life-course SEP, the same covariates, especially education level, confounded both associations.

The results of the sensitivity analysis demonstrated that the distribution of all variables significantly differed between the analytical and excluded samples (Supplementary Table 3). Cases of MCI were greater in the analytical sample than in the excluded sample. However, not having MCI was characteristic to most participants in both groups. In addition, a higher proportion of individuals were of a low SEP at birth in the excluded sample. At age 42, the majority of respondents were of a high SEP in both samples, but the analytical sample had a larger proportion that made up this category. In terms of life-course SEP, a greater percentage of participants remained at a high SEP or moved upward in the socioeconomic hierarchy within the analytical sample. In contrast, predominantly more individuals moved upward or remained at a low SEP within the excluded sample.

The excluded sample consisted of a greater percentage of females. It also had a higher number of individuals who were single and had no academic qualifications compared to the analytical sample. In terms of health behaviours, a slightly higher proportion of individuals in the excluded sample were current smokers and never consumed fruit and vegetables. The analytical sample had more participants, and thus, larger proportions of respondents who were physically active consumed alcohol on a weekly basis and ate fruit and vegetables daily. Finally, the analytical sample had a higher proportion of individuals with a physical health condition, but the excluded sample had a higher mean Malaise Inventory score.

## Discussion

In this nationally representative longitudinal population study, we observed an inverse association between life-course SEP and MCI onset in later life, independent of demographic, health behaviour, and health status covariates. Participants who moved upward, downward, or remained at a low SEP throughout their lives were significantly associated with higher odds of MCI onset in later life compared to those of a high SEP across the life course, independent of an important number of relevant covariates. The effect size for those who moved upward in SEP was smaller than for individuals who moved downward or were of a low life-course SEP. The odds of MCI development were also significantly higher when SEP was assessed separately in childhood and adulthood.

Possible explanations for the findings observed between the various life-course SEP backgrounds and MCI are that participants who were always of a low SEP from birth to age 42 may not have the financial resources to support a lifestyle associated with a reduced risk of developing cognitive impairment in later life [5]. This stems from SEP being heavily intertwined with several different risk factors associated with cognitive impairment, such as engaging in unhealthy behaviours or limited education opportunities, in addition to having poor social networks and support [5, 27]. This is supported by the present study’s findings that education and higher fruit and vegetable intake were strong predictors of MCI onset. Socioeconomic disadvantage is also correlated with a higher risk of cardiovascular disease, hypertension, type 2 diabetes in women, and depression, all of which are strong risk factors for both MCI and dementia [7, 28–38]. Similarly, the present data indicated that psychiatric symptoms were a strong confounder.

Specifically looking at the mobility directions, moving upward in SEP may be associated with greater odds of MCI formation in later life to a lesser extent than moving downwards due to experiencing a protective effect from being of a higher SEP during adulthood [5]. This protective effect may serve as a buffer against the adverse effects of childhood disadvantage on the risk of MCI [5]. For individuals who moved downward in SEP later in life, it is speculated that the protective effects induced by being born into a high SEP are weakened by the adverse effects associated with being of a lower SEP in adulthood, suggesting that SEP at adulthood may have a larger impact on MCI onset than childhood SEP [5]. We recommend further exploration of the mechanisms behind how childhood and adulthood SEP interact and differ in their cumulative impact on cognitive impairment.

The present study identified SEP as a strong predictor of MCI. The incorporation of a life-course approach when assessing SEP produced results that are consistent with recent findings despite the current literature being limited and solely looking at dementia [5, 39]. As Hazzouri and colleagues observed, participants of the present study who moved upward or downward in SEP were associated with higher odds of MCI compared to those who were always of a high SEP [5]. Of the two mobility directions, moving downward in SEP was similarly associated with greater odds of MCI formation than moving upward [5]. Looking at SEP separately at two different life stages, the results are also in line with recent publications that found significantly higher odds of MCI associated with low SEP during early life, such as at birth or childhood, and adulthood relative to those of a high SEP [2–4, 40].

### Strengths and Limitations

To the best of current knowledge, this is the first study to examine the relationship between life-course SEP and MCI amongst older adults monitored over their entire lives as part of the 1958 British Birth cohort. Examining this study cohort is beneficial in providing rich data collected from birth to older ages to better observe and understand how individuals’ SEP can vary over time and differentially impact cognitive health in later life. Its longitudinal cohort design reduced the likelihood of reverse causation from occurring as the outcome, MCI, was assessed years after measuring SEP at two time points to evaluate life-course SEP. In addition, several different covariates were able to be included in the analyses due to the large amount of data collected through comprehensive surveys at multiple sweeps. This allowed for comprehensive analyses to be conducted. Furthermore, the results of these analyses can be applied at a national level due to the utilization of a relatively large sample size obtained from the population of Great Britain.

Limitations of the present study were that participants were relatively young at the time of MCI identification. The likelihood of developing MCI is much greater with older age [7, 17]. In turn, fewer MCI cases may have been present, and thus the data may not have adequately represented the true association between life-course SEP and MCI. Furthermore, the loss of participants and exclusion criteria may have resulted in attrition and selection bias, in addition to a reduction in statistical power. Although we conducted a sensitivity analysis, the analytical and excluded samples demonstrated significant differences in variable distribution. Thus, the observed results may not be entirely representative of the study population. We also were unable to assess all covariate data from the same sweep. This may have affected the present study’s findings to an uncertain extent, as data limitations hindered supplementary sensitivity analyses. In addition, approximately 97% of cohort members were ethnically White British or Irish. Therefore, the results may not be generalizable to individuals of different ethnicities living in Great Britain. Finally, we cannot infer causality because residual confounding factors unaccounted for may have impacted the observed results.

## Conclusions

Using a prospective cohort design, the present study demonstrated strong evidence of an inverse association between life-course SEP and MCI onset in later life, in which lower life-course SEP was significantly associated with higher odds of MCI onset amongst middle-aged NCDS cohort members. These findings are important as such an association has not been priorly assessed in the general population in Great Britain. They also contribute to the limited literature regarding the significant correlation between life-course SEP and MCI. Future research should incorporate a life-course aspect regarding SEP to better establish its effects on cognitive health and investigate the underlying mechanisms associated with SEP throughout the life course, particularly the unique effects of SEP mobility. In addition, public health strategies should encompass a life-course approach when designing interventions to best address different determinants of impaired cognition that may be more prominent at different life stages.

### Supplementary Information

Below is the link to the electronic supplementary material.

Supplementary file1 (DOCX 45 KB)

## Data Availability

The data sets generated and analysed for the present study are available through the UK Data Services and freely available to researchers worldwide. The NCDS data can be accessed here: https://discover.ukdataservice.ac.uk.

## Abbreviations

SEP: Socioeconomic position
MCI: Mild cognitive impairment
NCDS: National Child Development Study
SD: Standard deviation
CI: Confidence interval
