# Supplementary material for: Life-Course Socioeconomic Position and Mild Cognitive Impairment in Midlife: Evidence from the 1958 British Birth Cohort
Source: J Epidemiol Glob Health. 2024 Jan 8;14(1):102–10. doi: 10.1007/s44197-023-00173-6 (PMC11043258; doi:10.1007/s44197-023-00173-6)
Supplement: Supplementary file 1 — Supplementary file1 (DOCX 45 KB) [file 44197_2023_173_MOESM1_ESM.docx]

Supplementary Information:

European Journal of Epidemiology and Global Health

Life-course Socioeconomic Position and Mild Cognitive Impairment in Midlife: Evidence from the 1958 British Birth Cohort

Chanthie Menika Sahota^1^, MSc; Noriko Cable^1^, PhD; Dorina Cadar, PhD^2,3,4^

^1^ Department of Epidemiology and Public Health, University College London, London, UK

^2^ Department of Behavioural Science and Health, University College London, London, UK

^3^ Department of Neuroscience, Brighton and Sussex Medical School, Sussex, UK

^4^ Department of Primary Care, Brighton and Sussex Medical School, Sussex, UK

Corresponding author:

Dr Dorina Cadar

Department of Neuroscience,

Brighton and Sussex Medical School,

Trafford Centre, BN1 9RH

Sussex, UK

Email: d.cadar@bsms.ac.uk

Supplementary Tables

Supplementary Table 1. Results of the logistic regressions for SEP at birth and MCI in midlife in the analytical sample from the 1958 British Birth Cohort

| **Variable** | **Model 1** | | | **Model 2** | | | **Model 3** | | | **Model 4** | | |
| --- | --- | --- | --- | --- | --- | --- | --- | --- | --- | --- | --- | --- |
|  | **OR** | **95% CI** | **p-value** | **OR** | **95% CI** | **p-value** | **OR** | **95% CI** | **p-value** | **OR** | **95% CI** | **p-value** |
| SEP at birth |  |  |  |  |  |  |  |  |  |  |  |  |
| High | 1 | - | - | 1 | - | - | 1 | - | - | 1 | - | - |
| Low | 1.73 | 1.48-2.01 | <0.001 | 1.33 | 1.13-1.56 | <0.001 | 1.28 | 1.09-1.50 | 0.003 | 1.27 | 1.08-1.50 | 0.003 |
| Sex |  |  |  |  |  |  |  |  |  |  |  |  |
| Male | 1 | - | - | 1 | - | - | 1 | - | - | 1 | - | - |
| Female | 0.68 | 0.59-0.79 | <0.001 | 0.70 | 0.61-0.81 | <0.001 | 0.69 | 0.59-0.80 | <0.001 | 0.67 | 0.57-0.78 | <0.001 |
| Marital status |  |  |  |  |  |  |  |  |  |  |  |  |
| Married | 1 | - | - | 1 | - | - | 1 | - | - | 1 | - | - |
| Cohabiting | 1.05 | 0.82-1.35 | 0.686 | 1.03 | 0.80-1.33 | 0.791 | 1.02 | 0.79-1.32 | 0.875 | 1.02 | 0.79-1.31 | 0.890 |
| Single | 1.30 | 1.01-1.67 | 0.044 | 1.34 | 1.03-1.73 | 0.028 | 1.30 | 1.00-1.69 | 0.048 | 1.29 | 0.99-1.67 | 0.055 |
| Divorced/sep/wid | 1.17 | 0.92-1.48 | 0.207 | 1.09 | 0.86-1.39 | 0.476 | 1.06 | 0.83-1.35 | 0.645 | 1.05 | 0.82-1.35 | 0.681 |
| Education level |  |  |  |  |  |  |  |  |  |  |  |  |
| No quals |  |  |  | 1 | - | - | 1 | - | - | 1 | - | - |
| Intermediate  quals |  |  |  | 0.52 | 0.43-0.62 | <0.001 | 0.56 | 0.47-0.67 | <0.001 | 0.57 | 0.48-0.69 | <0.001 |
| Higher quals |  |  |  | 0.17 | 0.13-0.22 | <0.001 | 0.20 | 0.15-0.26 | <0.001 | 0.21 | 0.16-0.27 | <0.001 |
| Smoking status |  |  |  |  |  |  |  |  |  |  |  |  |
| Never |  |  |  |  |  |  | 1 | - | - | 1 | - | - |
| Ex-smoker |  |  |  |  |  |  | 0.92 | 0.77-1.10 | 0.353 | 0.91 | 0.76-1.09 | 0.291 |
| Current |  |  |  |  |  |  | 0.96 | 0.80-1.14 | 0.623 | 0.95 | 0.79-1.13 | 0.547 |
| Physically active |  |  |  |  |  |  |  |  |  |  |  |  |
| No |  |  |  |  |  |  | 1 | - | - | 1 | - | - |
| Yes |  |  |  |  |  |  | 0.83 | 0.70-0.97 | 0.019 | 0.83 | 0.71-0.98 | 0.027 |

Supplementary Table 1. Cont’d results of the logistic regressions for SEP at birth and MCI in midlife in the analytical sample from the 1958 British Birth Cohort

| Alcohol consumption |  |  |  |  |  |  |  |  |  |  |  |  |
| --- | --- | --- | --- | --- | --- | --- | --- | --- | --- | --- | --- | --- |
| Never/infreq |  |  |  |  |  |  | 1 | - | - | 1 | - | - |
| Monthly |  |  |  |  |  |  | 0.94 | 0.75-1.17 | 0.582 | 0.95 | 0.76-1.19 | 0.678 |
| Weekly |  |  |  |  |  |  | 0.81 | 0.67-0.98 | 0.029 | 0.82 | 0.68-1.00 | 0.046 |
| Fruit & veg intake |  |  |  |  |  |  |  |  |  |  |  |  |
| Daily |  |  |  |  |  |  | 1 | - | - | 1 | - | - |
| 3-6 days/week |  |  |  |  |  |  | 0.65 | 0.50-0.84 | 0.001 | 0.64 | 0.49-0.84 | 0.001 |
| 2 or less days/week |  |  |  |  |  |  | 1.41 | 1.20-1.66 | <0.001 | 1.38 | 1.18-1.62 | <0.001 |
| Never |  |  |  |  |  |  | 1.69 | 1.10-2.62 | 0.018 | 1.64 | 1.06-2.53 | 0.027 |
| Physical health cond |  |  |  |  |  |  |  |  |  |  |  |  |
| No |  |  |  |  |  |  |  |  |  | 1 | - | - |
| Yes |  |  |  |  |  |  |  |  |  | 1.06 | 0.86-1.32 | 0.579 |
| Malaise Inventory score |  |  |  |  |  |  |  |  |  | 1.03 | 1.01-1.06 | 0.002 |

Model 1 adjusted for sex and marital status; Model 2 adjusted for Model 1 covariates and education level; Model 3 adjusted for Model 2 covariates, smoking status, physical activity, alcohol consumption, and fruit and vegetable intake; Model 4 adjusted for Model 3 covariates, physical health conditions, and psychiatric symptoms.

Supplementary Table 2. Results of the logistic regressions for adulthood SEP and MCI in midlife in the analytical sample from the 1958 British Birth Cohort

| **Variable** | **Model 1** | | | **Model 2** | | | **Model 3** | | | **Model 4** | | |
| --- | --- | --- | --- | --- | --- | --- | --- | --- | --- | --- | --- | --- |
|  | **OR** | **95% CI** | **p-value** | **OR** | **95% CI** | **p-value** | **OR** | **95% CI** | **p-value** | **OR** | **95% CI** | **p-value** |
| SEP at age 42 |  |  |  |  |  |  |  |  |  |  |  |  |
| High | 1 | - | - | 1 | - | - | 1 | - | - | 1 | - | - |
| Low | 2.28 | 1.98-2.63 | <0.001 | 1.65 | 1.42-1.92 | <0.001 | 1.58 | 1.35-1.84 | <0.001 | 1.57 | 1.35-1.84 | <0.001 |
| Sex |  |  |  |  |  |  |  |  |  |  |  |  |
| Male | 1 | - | - | 1 | - | - | 1 | - | - | 1 | - | - |
| Female | 0.78 | 0.67-0.90 | 0.001 | 0.76 | 0.66-0.88 | <0.001 | 0.75 | 0.64-0.87 | <0.001 | 0.72 | 0.62-0.84 | <0.001 |
| Marital status |  |  |  |  |  |  |  |  |  |  |  |  |
| Married | 1 | - | - | 1 | - | - | 1 | - | - | 1 | - | - |
| Cohabiting | 1.05 | 0.82-1.34 | 0.721 | 1.03 | 0.80-1.33 | 0.809 | 1.02 | 0.79-1.32 | 0.877 | 1.02 | 0.79-1.32 | 0.880 |
| Single | 1.27 | 0.98-1.63 | 0.070 | 1.30 | 1.01-1.69 | 0.044 | 1.28 | 0.99-1.66 | 0.062 | 1.27 | 0.98-1.65 | 0.071 |
| Divorced/sep/wid | 1.13 | 0.89-1.44 | 0.317 | 1.08 | 0.84-1.37 | 0.548 | 1.05 | 0.82-1.34 | 0.708 | 1.04 | 0.81-1.33 | 0.744 |
| Education level |  |  |  |  |  |  |  |  |  |  |  |  |
| No quals |  |  |  | 1 | - | - | 1 | - | - | 1 | - | - |
| Intermediate quals |  |  |  | 0.57 | 0.47-0.68 | <0.001 | 0.60 | 0.50-0.73 | <0.001 | 0.62 | 0.51-0.74 | <0.001 |
| Higher quals |  |  |  | 0.20 | 0.15-0.27 | <0.001 | 0.23 | 0.18-0.31 | <0.001 | 0.24 | 0.18-0.32 | <0.001 |
| Smoking status |  |  |  |  |  |  |  |  |  |  |  |  |
| Never |  |  |  |  |  |  | 1 | - | - | 1 | - | - |
| Ex-smoker |  |  |  |  |  |  | 0.90 | 0.75-1.08 | 0.245 | 0.89 | 0.74-1.06 | 0.197 |
| Current |  |  |  |  |  |  | 0.92 | 0.77-1.10 | 0.378 | 0.91 | 0.76-1.09 | 0.318 |
| Physically active |  |  |  |  |  |  |  |  |  |  |  |  |
| No |  |  |  |  |  |  | 1 | - | - | 1 | - | - |
| Yes |  |  |  |  |  |  | 0.84 | 0.72-0.99 | 0.040 | 0.85 | 0.73-1.00 | 0.054 |
| Alcohol consumption |  |  |  |  |  |  |  |  |  |  |  |  |
| Never/infrequently |  |  |  |  |  |  | 1 | - | - | 1 | - | - |
| Monthly |  |  |  |  |  |  | 0.97 | 0.77-1.21 | 0.761 | 0.98 | 0.79-1.22 | 0.865 |
| Weekly |  |  |  |  |  |  | 0.83 | 0.68-1.00 | 0.049 | 0.84 | 0.70-1.02 | 0.074 |

Supplementary Table 2. Cont’d results of the logistic regressions for adulthood SEP and MCI in midlife in the analytical sample from the 1958 British Birth Cohort

| Fruit & veg intake |  |  |  |  |  |  |  |  |  |  |  |  |
| --- | --- | --- | --- | --- | --- | --- | --- | --- | --- | --- | --- | --- |
| Daily |  |  |  |  |  |  | 1 | - | - | 1 | - | - |
| 3-6 days/week |  |  |  |  |  |  | 0.67 | 0.51-0.87 | 0.002 | 0.66 | 0.51-0.86 | 0.002 |
| 2 or less days/week |  |  |  |  |  |  | 1.42 | 1.21-1.67 | <0.001 | 1.39 | 1.18-1.63 | <0.001 |
| Never |  |  |  |  |  |  | 1.68 | 1.08-2.60 | 0.020 | 1.62 | 1.05-2.51 | 0.031 |
| Physical health cond |  |  |  |  |  |  |  |  |  |  |  |  |
| No |  |  |  |  |  |  |  |  |  | 1 | - | - |
| Yes |  |  |  |  |  |  |  |  |  | 1.06 | 0.85-1.31 | 0.620 |
| Malaise Inventory score |  |  |  |  |  |  |  |  |  | 1.03 | 1.01-1.06 | 0.002 |

Model 1 adjusted for sex and marital status; Model 2 adjusted for Model 1 covariates and education level; Model 3 adjusted for Model 2 covariates, smoking status, physical activity, alcohol consumption, and fruit and vegetable intake; Model 4 adjusted for Model 3 covariates, physical health conditions, and psychiatric symptoms.

Supplementary Table 3. Descriptive characteristics of the excluded and analytical samples from the 1958 British Birth Cohort

| **Variable** | **Category** | **Excluded Sample**  **(N=11,968)** | **Analytical Sample**  **(N=6590)** | **p-value** |
| --- | --- | --- | --- | --- |
| MCI status | No | 2276 (81.4%) | 5661 (85.9%) | <0.001 |
|  | Yes | 520 (18.6%) | 929 (14.1%) |  |
| SEP at birth | High | 3288 (28.8%) | 2448 (37.2%) | <0.001 |
|  | Low | 8136 (71.2%) | 4142 (62.9%) |  |
| SEP at age 42 | High | 1765 (58.8%) | 4394 (66.7%) | <0.001 |
|  | Low | 1237 (41.2%) | 2196 (33.3%) |  |
| Life-course SEP | Always high | 637 (22.7%) | 1979 (30.0%) | <0.001 |
|  | Upward | 995 (35.5%) | 2415 (36.7%) |  |
|  | Downward | 205 (7.3%) | 469 (7.1%) |  |
|  | Always low | 969 (34.5%) | 1727 (26.2%) |  |
| Sex | Male | 2267 (47.0%) | 3359 (51.0%) | <0.001 |
|  | Female | 2562 (53.1%) | 3231 (49.0%) |  |
| Marital status | Married | 3114 (65.3%) | 4933 (74.9%) | <0.001 |
|  | Cohabiting | 469 (9.8%) | 577 (8.8%) |  |
|  | Single | 522 (11.0%) | 486 (7.4%) |  |
|  | Divorced/separated/widowed | 662 (13.9%) | 594 (9.0%) |  |
| Education level | No qualifications | 1104 (34.6%) | 806 (12.2%) | <0.001 |
|  | Intermediate qualifications | 1409 (44.1%) | 4105 (62.3%) |  |
|  | Higher qualifications | 681 (21.3%) | 1679 (25.5%) |  |
| Smoking status | Never | 1914 (40.0%) | 3159 (47.9%) | <0.001 |
|  | Ex-smoker | 1135 (23.7%) | 1734 (26.3%) |  |
|  | Current | 1736 (36.3%) | 1697 (25.8%) |  |
| Physically active | No | 1352 (28.3%) | 1593 (24.2%) | <0.001 |
|  | Yes | 3431 (71.7%) | 4997 (75.8%) |  |
| Alcohol consumption | Never/infrequently | 1182 (24.7%) | 1242 (18.9%) | <0.001 |
|  | Monthly | 891 (18.7%) | 1331 (20.2%) |  |
|  | Weekly | 2705 (56.6%) | 4017 (61.0%) |  |
| Fruit & veg intake | Daily | 2384 (49.8%) | 3548 (53.8%) | <0.001 |
|  | 3-6 days/week | 557 (11.7%) | 892 (13.5%) |  |
|  | 2 or less days/week | 1660 (34.7%) | 2026 (30.7%) |  |
|  | Never | 182 (3.8%) | 124 (1.9%) |  |

Supplementary Table 3. Cont’d descriptive characteristics of the excluded and analytical samples from the 1958 British Birth Cohort

| Physical health condition | No | 4107 (85.9%) | 5833 (88.5%) | <0.001 |
| --- | --- | --- | --- | --- |
|  | Yes | 675 (14.1%) | 757 (11.5%) |  |
| Malaise Inventory score | Mean (SD) | 4.18 (4.15) | 3.18 (3.14) | <0.001 |
